# CO1 barcodes resolve an asymmetric biphyletic clade for *Diabrotica undecimpunctata* subspecies and provide nucleotide variants for differentiation from related lineages using real-time PCR

**DOI:** 10.3389/finsc.2023.1168586

**Published:** 2023-04-20

**Authors:** Luke R. Tembrock, Christina R. Wilson, Frida A. Zink, Alicia E. Timm, Todd M. Gilligan, Alexander S. Konstantinov, Alexey K. Tishechkin

**Affiliations:** ^1^Department of Agricultural Biology, Colorado State University, Fort Collins, CO, United States; ^2^Pest Identification Technology Laboratory, USDA-APHIS-PPQ-Science and Technology, Fort Collins, CO, United States; ^3^Systematic Entomology Laboratory, USDA-ARS, Washington, DC, United States; ^4^Plant Pest Diagnostics Branch, California Department of Food and Agriculture, Sacramento, CA, United States

**Keywords:** spotted cucumber beetle, maternal inheritance, species concept, plant pathogen vector, ITS2, *Diabrotica virgifera*, monophyly

## Abstract

*Diabrotica undecimpunctata* is a multivoltine polyphagous beetle species that has long been documented as a significant agricultural pest throughout its native range in North America. This beetle can vector bacterial and viral plant pathogens that result in major losses to crops such as cucumber and soybean. Many countries outside the Americas treat *D. undecimpunctata* as a species of quarantine importance, while in the USA only the subspecies *D. u. duodecimnotata* is subject to quarantine, to prevent introduction from Mexico. Identification of *D. undecimpunctata* on the basis of morphology alone can be complicated given the use of conflicting characters in the description of some subspecific taxa. To better understand relationships among *D. undecimpunctata* subspecies and other related species, we sequenced mitochondrial cytochrome oxidase 1 (CO1) and nuclear internal transcribed spacer 2 (ITS2) DNA from individuals in different subspecific taxa and across different parts of the species range using museum samples and interceptions. When our data were combined with publicly available *Diabrotica* data, no pattern of divergence consistent with the currently recognized subspecific designations was found. In addition, we compared phylogenetic patterns in CO1 data from the congener *D. virgifera* to demonstrate the utility of mitochondrial data in resolving subspecies. From the CO1 data, a diagnostic real-time PCR assay was developed that could successfully identify all haplotypes within the large *D. undecimpunctata* clade for use in surveys and identification at ports of entry. These findings underscore the need to resolve molecular and morphological datasets into cogent, lineage-based groupings. Such efforts will provide an evolutionary context for the study of agriculturally important attributes of *Diabrotica* such as host preferences, xenobiotic metabolism, and natural and anthropogenic patterns of dispersal.

## 1 Introduction

The leaf beetle species *Diabrotica undecimpunctata* Mannerheim (Coleoptera, Chrysomelidae) has been documented as a serious pest of agricultural importance in North America for well over 100 years (1–3). Adult *D. undecimpunctata* feed on more than 50 plant species, including food, feed, and fiber crops such as alfalfa, apple, beans, beets, cotton, cucurbits, hemp, maize, peach, peas, potato, sunflower, and tomato, as well as causing damage to ornamentals such as canna, carnation, dahlia, peony, rose, and wisteria (4–7). The larvae are root feeders and are often found on maize roots but have also been frequently noted on other species, including several grass species, alfalfa, and peas (5, 7, 8). While the economic impact of *D. undecimpunctata* is less than the US$1 billion annual estimate associated with *D. virgifera* (9), *D. undecimpunctata* does account for serious economic losses during large infestations (6, 10, 11). One of the most problematic aspects of *D. undecimpunctata* feeding is the transmission of the virulent bacterial pathogen *Erwinia tracheiphila* (12, 13), which can cause rapid crop failure among cucurbitaceous species (14). In addition to transmission of *E. tracheiphila*, *D. undecimpunctata* is a competent vector of the bean pod mottle virus, which can cause losses in soybean (15). On account of these concerns and the potential for establishment outside the historic species range (16), *D. undecimpunctata* was recently categorized as an A1 quarantine pest under Annex IIA by the Panel on Plant Health of the European Food Safety Authority (17).

Given the importance of *D. undecimpunctata* to agriculture, it has been the subject of several taxonomic revisions [summarized in (18)]. The currently accepted taxonomic arrangement of *D. undecimpunctata* subdivides the species into four subspecies based primarily on morphological differences (19): *D. u. undecimpunctata* Mannerheim 1843, *D. u. duodecimnotata* Harold 1875, *D. u. howardi* Barber 1947, and *D. u. tenella* LeConte 1858. Difficulty in differentiating between *D. undecimpunctata* subspecies, such as *D. u. howardi* and *D. u. tenella*, on the basis of morphology has been previously noted [e.g. (20)]. Some molecular phylogenetic work has been conducted in *Diabrotica* and related lineages, but much of this has been at the genus level and above, with very little at the subspecies level (21–23). Generating DNA sequence data sampled at the subspecific level for *D. undecimpunctata* will provide a genetic genealogical history that pertains to subspecific designations for this group and will ultimately improve taxonomic, agronomic, and phylogeographic studies.

Historically, *Diabrotica* has been split into the three species groups *fucata*, *signifera*, and *virgifera* (24). While less well studied than the spread of species in the *virgifera* species group, it is thought that the expansion and establishment of *D. undecimpunctata* in North American agroecosystems has also been the result of human activities, such as the large-scale cultivation of maize, cucurbits, and other host plants, as well as trade in agricultural commodities (25–27). To this point, *D. u. duodecimnotata* is frequently intercepted on agricultural commodities imported from Mexico into the USA during border inspections. The subspecies *D. u. duodecimnotata* is considered a quarantine pest by the United States Department of Agriculture (USDA) based on its potential to cause damage to agricultural crops. *Diabrotica* beetles have also been a concern outside the Americas since the introduction of *D. virgifera virgifera* to Europe in 1992, with its subsequent impact on agricultural production (28, 29). Given the limited molecular data sampled at the subspecific level for *D. undecimpunctata* and the concerns regarding introduction and establishment outside its native range, we generated mitochondrial cytochrome oxidase 1 (CO1) and nuclear 45S internal transcribed spacer 2 (ITS2) sequence data from intercepted and museum specimens to address the following questions: (1) can current or novel subspecies of *D. undecimpunctata* be recognized *via* CO1 and/or ITS2 sequence data? and (2) do CO1 data provide fixed nucleotide variants for the development of a reliable real-time PCR assay to separate all subspecies of *D. undecimpunctata* from closely related species?

## 2 Materials and methods

### 2.1 Sample acquisition

From 2018 to 2019, 254 *Diabrotica* beetles were identified at ports of entry coming from Mexico into the USA. Of these, 115 samples were preserved in absolute ethanol for later study. In addition to these beetles acquired from interceptions, loans of *Diabrotica* beetles were made from the Smithsonian National Museum of Natural History, the American Museum of Natural History, the California Department of Food and Agriculture, and the University of California at Berkeley and Davis. The 18 most recently collected museum specimens, mainly from the Smithsonian, were sampled for non-destructive DNA extraction and sequencing. Specimens used are summarized in **Table 1** and sample details for sequences generated in this study can be found using GenBank accessions OQ649623–OQ649739 for CO1 and OQ641612–OQ641622 for ITS2.

**Table 1:** Samples used to resolve maternal relationships among *Diabrotica* species and for development and/or testing of a real-time PCR assay.

Species	Origin	Sample type	Quantity
*Diabrotica adelpha*	N/A	NCBI	2
*Diabrotica amecameca*	N/A	NCBI	3
*Diabrotica balteata*	N/A	NCBI	3
*Diabrotica balteata*	USA/Mexico border	Intercept Sanger	2*
*Diabrotica balteata*	Mexico	Museum Sanger	1
*Diabrotica barberi*	N/A	BOLD	1
*Diabrotica barberi*	N/A	NCBI	25
*Diabrotica biannularis*	N/A	NCBI	1
*Diabrotica cristata*	N/A	BOLD	12
*Diabrotica cristata*	N/A	NCBI	2
*Diabrotica decempunctata*	N/A	NCBI	1
*Diabrotica dissimilis*	N/A	NCBI	1
*Diabrotica limitata*	N/A	NCBI	2
*Diabrotica longicornis*	N/A	NCBI	1
*Diabrotica nummularis*	N/A	NCBI	1
*Diabrotica porracea*	N/A	NCBI	3
*Diabrotica scutellata*	N/A	NCBI	1
*Diabrotica sexmaculata*	N/A	NCBI	1
*Diabrotica speciosa*	N/A	NCBI	5
*Diabrotica tibialis*	N/A	NCBI	2
*Diabrotica undecimpunctata duodecimnotata*	USA/Mexico border	Intercept Sanger	90*
*Diabrotica undecimpunctata duodecimnotata*	Mexico	Museum Sanger	1
*Diabrotica undecimpunctata howardi*	N/A	NCBI	2
*Diabrotica undecimpunctata howardi*	Mexico, USA (KY, MN, MO, OH, SC)	Museum Sanger	6
*Diabrotica undecimpunctata tenella*	USA (AZ, NV, TX, UT)	Museum Sanger	4
*Diabrotica undecimpunctata undecimpunctata*	USA (AZ, CA, NM, OR)	Museum Sanger	5*
*Diabrotica undecimpunctata undecimpunctata*	N/A	NCBI	1
*Diabrotica undecimpunctata* ssp.	Mexico	Museum Sanger	1
*Diabrotica undecimpunctata* ssp.	USA/Mexico border	Intercept Sanger	7
*Diabrotica undecimpunctata* ssp.	USA (AR, AZ, FL)	BOLD	8
*Diabrotica undecimpunctata* ssp.	N/A	NCBI	9
*Diabrotica virgifera*	N/A	BOLD	1
*Diabrotica virgifera*	N/A	NCBI	14
*Diabrotica viridula*	N/A	NCBI	4
*Diabrotica wartensis*	N/A	NCBI	3
*Lyctus africanus*	USA (NY intercepted)	Intercept Sanger	1
*Trichobrotica nymphaea*	N/A	NCBI	1

Sanger sequencing was used to generate CO1 and ITS2 sequences from intercepts and museum samples. *Sample sets from which ITS2 sequences were generated. N/A, not applicable.

### 2.2 DNA extraction

DNA was extracted from individual adult specimens of *Diabrotica* preserved in ethanol or from pinned museum specimens using a Lucigen MasterPure DNA extraction kit (Lucigen Corp., Middleton, WI, USA). Isolation of DNA from intercepted specimens preserved in ethanol was preceded by drying the specimens in 1.5mL microcentrifuge tubes on a digital dry bath set to 55°C for 20 to 30 minutes. Museum specimens were removed from their pins. All individual, whole dried specimens were placed in 1.5mL microcentrifuge tubes, immersed in 300 μL of Tissue and Cell Lysis Solution and 1 μL of Proteinase K, and heated to 65°C overnight on an Eppendorf ThermoMixer FP (Eppendorf SE, Hamburg, Germany) at 500 rpm. After overnight incubation, specimens were removed from the lysis buffer, rinsed with absolute ethanol, and returned to pins (museum specimens) or ethanol (intercepted specimens). The remaining extraction was carried out following the manufacturer’s instructions with modifications as described by Zink et al. (30). After elution, DNA concentration and purity were measured for a 2μL sample using a NanoDrop 2000 v 1.6 spectrophotometer (Thermo Scientific, Wilmington, DE, USA). Two readings were taken to ensure machine consistency. Throughout these and the following steps, all equipment and materials were sanitized between steps and filter tips were used to handle any liquids containing DNA to prevent contamination. Negative controls were employed during all DNA extraction and PCR steps to test for contamination.

### 2.3 CO1 and ITS2 PCR amplification and sequencing

All PCRs were performed on a Bio-Rad C1000 Touch thermal cycler (Bio-Rad Laboratories, Inc., Hercules, CA, USA). In order to generate sequences that could be aligned to previous *Diabrotica* CO1 datasets (22, 31) we employed the universal primers S1718 (32) and Nancy (33; **Table 2**). An optimized PCR protocol was used due to the degraded nature of many of the samples. The 50 μL reactions contained 32.75 μL molecular grade H_2_O, 5.00 μL 10× Ex Taq buffer, 4.00 μL deoxynucleoside triphosphate (dNTP) mixture at 2.5 mM, 200 nM S1718 forward primer, 200 nM Nancy reverse primer, 1.25 mM MgCl_2_, 0.05 mg bovine serum albumin, 1 unit of TaKaRa Ex Taq HS polymerase (Takara Bio Inc., Shiga, Japan), and 1 μL of DNA template of varying concentration or autoclaved ddH_2_O for no-tissue controls. The thermocycler protocol included an initial denaturation step of 94°C for 3 min, followed by 40 cycles of 94°C for 20 s, 52°C for 20 s, 72°C for 30 s, and a final extension at 72°C for 5 min. A lid temperature of 105°C was maintained throughout all cycles. A subset of samples amplified for CO1 showing haplotypic diversity were also amplified for ITS2 so that mitochondrial relationships could be compared against nuclear data. The general primers (located in 5.8S and 28S) and thermocycler protocol described in Navajas et al. (34) were used to generate complete ITS2-spanning amplicons for comparison with previously generated data (21); the reaction mixture employed was otherwise identical to that employed for the CO1 PCRs described above. Success of PCRs was confirmed on 1% agarose gels containing ethidium bromide and imaged with a UV light source (Analytik Jena, LLC, Jena, Germany). Reactions with visible bands were purified using a Qiagen QIAquick PCR purification kit following the manufacturer’s instructions (Qiagen Inc., Hilden, Germany). After purification, CO1 PCR products were sequenced at the University of Chicago Comprehensive Cancer Center DNA Sequencing Facility on an Applied Biosystems 3730XL DNA sequencer (Applied Biosystems, Foster City, CA, USA) using the same primers as for amplification. The ITS2 amplicons were sequenced with a 3730XL by Genewiz (Azenta Life Sciences Inc., Chelmsford, MA, USA). Sequences were manually trimmed to remove poor-quality base calls from the 5′ and 3′ ends (including primer sequences), assembled into contigs, and converted into consensus sequences in Geneious Prime 2021.0.3^1^ for use in subsequent analyses.

**Table 2:** Primers and probes used for this study.

Name	Use	Sequence	TM (°C)	Source
LC1	ITS2 PCR and sequencing	5′-GGGTCGATGAAGAACGCAGC	62.5*	34
HC2	ITS2 PCR and sequencing	5′-ATATGCTTAAATTCAGCGGG	54.3*	34
S1718	CO1 PCR and sequencing	5′-GGAGGATTTGGAAATTGATTAGTTCC	62.9*	32
Nancy	CO1 PCR and sequencing	5′-CCCGGTAAAATTAAAATATAAACTTC	58.4*	33
Dia_und_CO1_500F	Real-time PCR diagnostic	5′-ACAGTAATTAATATACGTCCAATAGGA	60.8	This study
Dia_und_CO1_600R	Real-time PCR diagnostic	5′-GGTAATGATAATAGTAGTAAAACTGCT	60.8	This study
Dia_und_CO1_535P	Real-time PCR diagnostic	5′-/56-FAM/TGACCGAAT/ZEN/ACCATTATTTGTATGAGCA/3IABkFQ/	64.4	This study
Chrys_18S_1981F	Real-time PCR control	5′-GGAAAGATGACCAAACTTGATC	58.4	This study
Chrys_18S_2066R	Real-time PCR control	5′-CTGTAATGATCCTTCCGCAG	58.4	This study
Chrys_18S_2016P	Real-time PCR control	5′-/5Cy5/AAAAGTCGT/TAO/AACAAGGTTTCCGT/3IAbRQSp/	59.2	This study

*indicates recalculated Tm from original source.

### 2.4 Phylogenetic and network analyses of DNA sequence data

The 117 consensus CO1 sequences generated in this study were combined with 85 *Diabrotica* sequences from GenBank and 22 from the Barcode of Life Data System (35). The combined dataset was aligned using MAFFT v 7.450 (36, 37) with default settings. Once aligned, the matrix was trimmed to exclude non-overlapping sequences to a size of 227 samples by 420 nucleotides and realigned using the same method as above. With the symmetrical matrix, a neighbor joining (NJ) distance tree was resolved using the Tamura-Nei distance model (38) with another chrysomelid in the same subtribe *Trichobrotica nymphaea* (AY242440) set as the outgroup and 1,000 jackknife replicates to assess branch support. Using MrBayes 3.2.6 (39), Bayesian inference (BI) was run with a general time reversible + invariable sites + gamma rate variation among sites (GTR+I+G) substitution and rate variation model, four gamma categories, four heated chains, a chain length of 1,100,000, a subsampling frequency of 200, a burn-in of 100,000, and an unconstrained branch length prior. A second MrBayes run was conducted with the JC69 nucleotide substitution model (all other settings the same as above) to assess for any differences in tree topology and branch support when using a less complex substitution model. A subset of 10 *D. undecimpunctata* samples were selected for amplification of ITS2 based on CO1 haplotype diversity. The same steps as for analysis of CO1 sequences were followed for ITS2 but with a trimmed matrix of 26 samples by 530 loci (including gaps) and the use of *Acalymma vittatum* (AF278557) as an outgroup. A dataset downloaded from GenBank (318 samples by 617 nucleotides) of CO1 sequences from the two subspecies of *D. virgifera* was analyzed (along with three outgroup taxa) using the above techniques and was employed as a basis for comparison with *D. undecimpunctata* given the phylogenetic and taxonomic similarities between these lineages. In order to visually resolve informative single nucleotide variants (SNVs) between CO1 haplotypes most closely related to *D. undecimpunctata* (based on the BI tree), the original trimmed matrix was reduced to a 156 sample by 420 nucleotide matrix and analyzed with the TCS statistical parsimony algorithm (40) as implemented in PopART 1.7 (41).

### 2.5 Diagnostic real-time PCR primer and probe design

The 227 sample by 420 nucleotide alignment of CO1 sequences (**Supplementary Data Sheet 1**) was used for primer and probe design to target *D. undecimpunctata* and exclude all other *Diabrotica* lineages. The alignment was manually scanned for regions of high SNV density with respect to differences outside *D. undecimpunctata* and low SNV density within *D. undecimpunctata* using consensus base calls as a guide. From the regions meeting these criteria, separate ~30-bp windows for forward primers, reverse primers, and a probe were selected within 100- to 150-bp segments of the alignment for automated primer and probe design. Five primers and probes were designed using Primer 3 v 2.3.7 (42) within each window with the following parameter settings for primers: monovalent salt 50 mM (divalent 1.5 mM); primer DNA 50 nM; dNTP 0.6 mM; Tm °C [calculated using the SantaLucia method (43)] min. 50, opt. 55.5, max. 60; GC% min. 20, opt. 50, max. 80; primer length nt min. 14, opt. 22, max. 35; max. dimer Tm 47; max. poly-X 5; and max. 3′ stability 9. All settings were the same for probe design except for: Tm °C min. 57, opt. 60, max. 63; GC % min. 20, opt. 50, max. 80; and probe length nt min. 18, opt. 20, max. 36. From the five primers and probes designed from each window, the best primers and probe were chosen based on the highest number of nucleotide variants with respect to other closely related species, location of the nucleotide variants (primer/probe sequence ends preferred), highest Tm differential between primers and probe, and lowest level of self-dimerization, hairpin formation, self-annealing, and hetero-dimerization to the other oligos. These structural and thermodynamic tests were conducted using the IDT OligoAnalyzer^2^ and/or OligoCalc (44). BLASTn searches of the entire nt database were also employed to ensure specificity of the selected primer and probe sequences in combination. Control primers and probe were also designed using the same parameters but with an 18S rDNA dataset retrieved from a BLASTn alignment constrained by ‘Coleoptera’. Manual optimization employed similarity instead of difference in window selection and lower Tm with respect to the diagnostic primers and probe as final selection criteria. Primer and probe designs were submitted to IDT (Integrated DNA Technologies, Coralville, IA, USA) for synthesis and purification. Final primer and probe sequences can be found in **Table 2**.

### 2.6 Duplex real-time PCR optimization and testing

The Minimum Information for Publication of Quantitative Real-time PCR Experiments (MIQE) guidelines (45) were followed wherever applicable. All optimization and testing processes were carried out on a Bio-Rad CFX96 Touch Real-time PCR Detection System (Bio-Rad Laboratories, Inc.) in 96-well, thin-walled, white-well, hard-shell PCR plates (Bio-Rad Laboratories, Inc.) sealed with optically clear Microseal ‘B’ seals (Bio-Rad Laboratories Inc.). The assay was optimized for annealing temperature and primer and probe concentration for both loci. The reaction mix used was as follows: 10.00 μL of 2× iTaq Universal Probe Supermix (Bio-Rad Laboratories, Inc.); control primers and probe of 500nM Chrys_18S_1981F, 500nM Chrys_18S_2066R, 250nM Chrys_18S_2016P (labeled with 56-FAM reporter); diagnostic primers and probe of 500nM Dia_und_500Fa, 500nM Dia_und_600R, 13.75nM Dia_und_CO1_535P (labeled with 5Cy5 reporter); 1.00 μL DNA template of varying concentration or ddH_2_O for no-tissue controls; and ddH_2_O to complete the dilution of the supermix to a final volume of 20.00 μL. The optimized thermocycler conditions were as follows: an initial denaturation step for 5 min at 95°C, followed by 40 cycles of 95°C for 15 s and 60°C for 15 s, followed by data capture. A lid temperature of 105°C was maintained throughout all cycles. The assay was tested on 128 individuals for two replicates conducted by different laboratory technicians on different days.

The sensitivity of the assay was tested with serial dilutions of *D. undecimpunctata* DNA at concentrations from 100 ng/µL to 0.0001 ng/µL using both the diagnostic and control probes in duplexed reactions. Results were averaged from four independent runs and the C_q_ values were compared to DNA concentration on a logarithmic scale to determine the slope, y-intercept, and correlation of DNA concentration to assay sensitivity (30, 46).

## 3 Results

**Figure 1:**
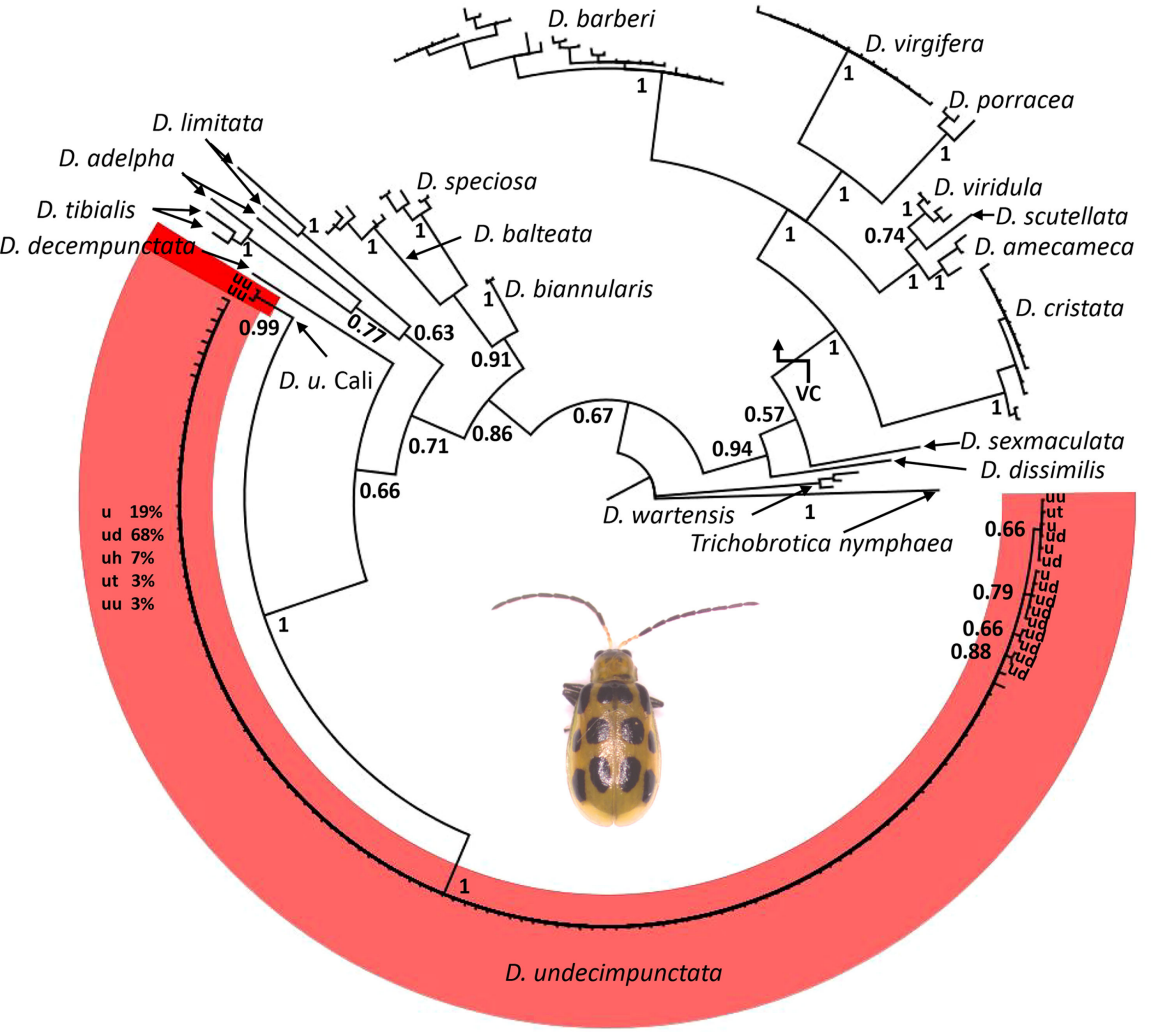
A phylogenetic tree for *Diabrotica* generated *via* Bayesian inference with a general time reversible (GTR) substitution model using an alignment of 227 CO1 DNA barcodes. Values at nodes are posterior probabilities; *Trichobrotica nymphaea* was set as an outgroup. Within *D. undecimpunctata*, subclade terminals are labeled by subspecies: *D. undecimpunctata* (u), *D. u. duodecimnotata* (ud), *D. u. howardi* (uh), *D. u. tenella* (ut), and *D. u. undecimpunctata* (uu). The large unstructured grade is labeled by percentage of terminals with a given subspecific designation. The *virgifera* species group is denoted VC in the tree, with all other *Diabrotica* terminals belonging to the *fucata* species group. Inset image is an adult *D. undecimpunctata*.

**Figure 2:**
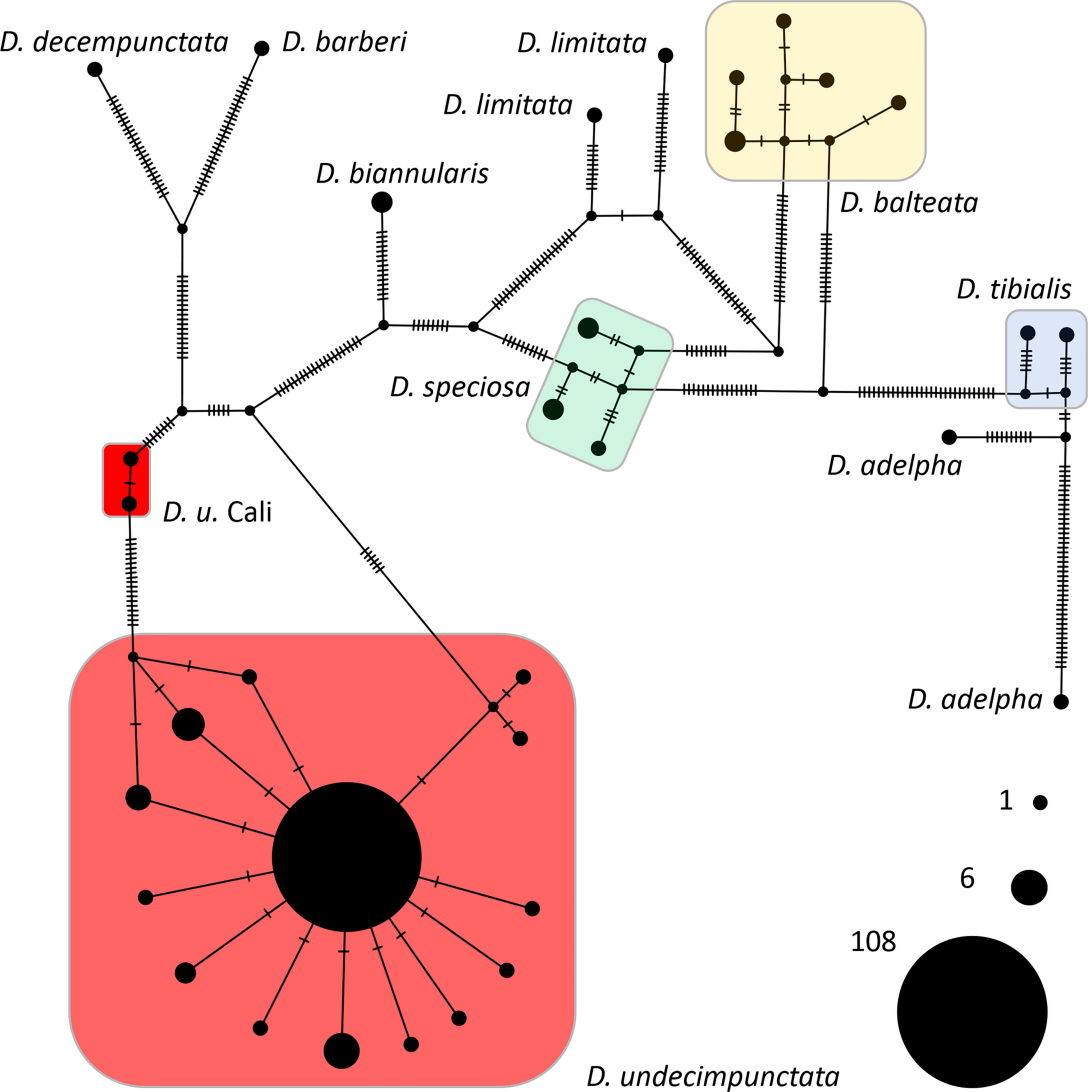
A TCS network from an alignment of 156 *Diabrotica* CO1 sequences. Each hatch mark along a branch indicates an informative nucleotide variant between haplotypes. Network terminals are scaled by the number of individuals sharing an identical haplotype; a key to the size of these terminals is provided in the lower right. The *D. undecimpunctata* haplogroups are color coded as in **Figure 1**.

From a combination of intercepted and museum chrysomelid specimens, we were able to generate 118 CO1 DNA barcodes for use in analyses and testing (**Table 1**). During the course of creation of sequence contigs and quality control, 10 specimens (not included in **Table 1**) were found to contain *Centistes* sp. (Braconidae) parasitoid wasp larvae [see (47)]. These specimens were excluded from phylogenetic analyses but were later used in testing the real-time PCR assay. From the CO1 alignment (**Supplementary Data Sheet 1**), the BI and NJ analyses (**Figure 1** and **Supplementary Figures 1–3**) resolved the *Diabrotica* species into two clades (*fucata* and *virgifera* species groups) and the *D. undecimpunctata* lineage (in the *fucata* group) into a well-supported monophyletic clade (posterior probability (PP) 1 GTR, PP 1 JC69, and NJ jackknife (JK) 99.8). Within the *D. undecimpunctata* clade, an early-diverging lineage containing two samples from western California was supported as distinct (PP 0.99 GTR, PP 1 JC69, and NJ JK 100). Network analysis of the subclade containing *D. undecimpunctata* and the most closely related species further confirmed a nearly homogeneous *D. undecimpunctata* and a next-nearest haplogroup containing the two western California specimens of *D. undecimpunctata* separated by 20 informative nucleotide variants along the shortest branch from the larger group (**Figure 2**). In comparison, for the large *D. undecimpunctata* haplogroup, excluding the western California samples, the longest path separating haplotypes was three nucleotide variants. Across this entire haplogroup, only 14 informative nucleotide variants of either one or two steps described all the haplotypic diversity. None of the *D. undecimpunctata* subspecies designations were isolated to a single haplogroup in the network analysis (**Figure 2**) or to any of the subclades in any of the BI trees (**Figure 1** and **Supplementary Figures 1, 2**) or NJ trees (**Supplementary Figure 3**). From the CO1 data, a subset of samples were selected to be sequenced for nuclear ITS2 to confirm (1) that the western California lineage was an outlier to other *D. undecimpunctata* samples, and (2) that the ingroup made up of different *D. undecimpunctata* subspecies was homogeneous. Samples for this test were selected from different haplotypic lineages (including the western California lineage) to maximize the possibility of finding ITS2 nucleotide variants. The results from ITS2 confirmed that the western California lineage was an outlier to all other *D. undecimpunctata* (**Supplementary Figures 4–6**) through the retention of an ancestral thymine at position 326 shared with all other *Diabrotica* species (**Supplementary Data Sheet 2**). The remaining *D. undecimpunctata* samples in the dataset were homogeneous in sequence except for an autapomorphic cytosine at 171 in *D. u. undecimpunctata* (AF278571) and an adenine at 430 in the same sample, which was a heterozygotic ‘R’ (adenine or guanine) peak in one intercepted *D. u. duodecimnotata* (DIA-007).

**Figure 3:**
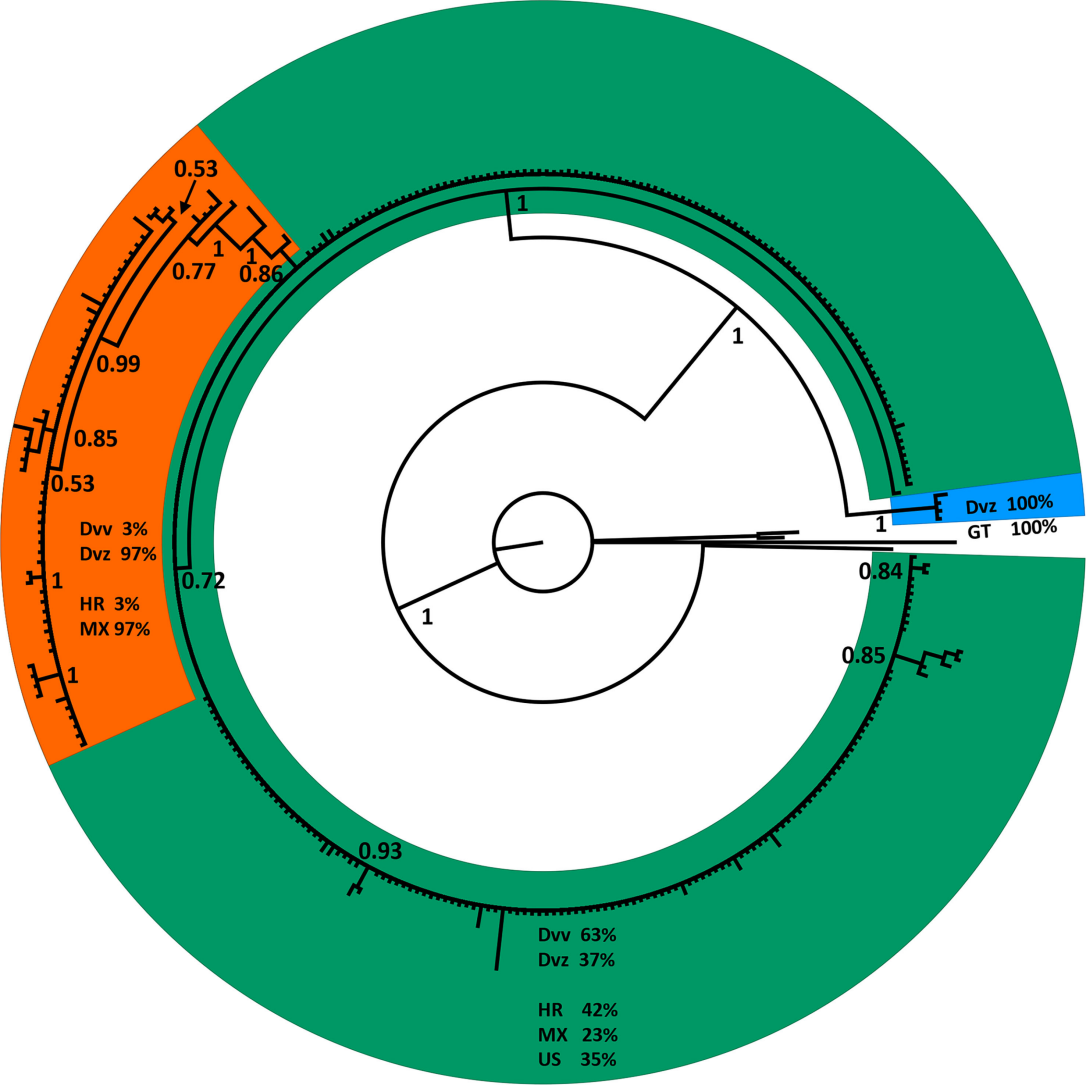
A phylogenetic tree generated *via* Bayesian inference with a general time reversible (GTR) substitution model using an alignment of CO1 barcodes for 314 *Diabrotica virgifera* identified to subspecies downloaded from GenBank. Values at nodes are posterior probabilities; one *D. undecimpunctata*, two *D. amecameca*, and one *D. porracea* were used as outgroup taxa. Clades are color coded as follows: orange, a mainly *D. v. zeae* clade from Mexico; green, a mixed *D. v. virgifera* and *D. v. zeae* clade from Mexico and the US; and blue, a *D. v. zeae-*only clade from Guatemala. For each colored clade, the percentage of terminals identified as *D. v. virgifera* (Dvv) and *D. v. zeae* (Dvz) is indicated, as well as the percentage of terminals from Guatemala (GT), Croatia (HR), Mexico (MX), and the United States (US).

The phylogenetic tree of *D. virgifera* subspecies, generated to assess the correlation of CO1 data to subspecific designations for use as a comparator to the congeneric *D. undecimpunctata* subspecies, resolved three well-supported clades from a CO1 alignment (**Supplementary Data Sheet 3**) across all tree-building methods (**Figure 3** and **Supplementary Figures 7–9**). The first lineage was early-diverging and consisted of four individuals from Guatemala, all identified as *D. v. zeae*. The second lineage contained individuals from Mexico (23%), the USA (35%), and Croatia (42%, which is inferred as the primary lineage from which the introduced populations were sourced), with individuals identified as both *D. v. virgifera* (63%) and *D. v. zeae* (37%). The third lineage consisted almost exclusively of individuals from Mexico (97%), along with a small number from Croatia (3%), with all Mexican samples identified as *D. v. zeae* and all Croatian samples as *D. v. virgifera*. Thus, the CO1 data from *D. virgifera* did not entirely correspond to subspecific designation but rather appeared to be associated with attributes such as unidirectional reproductive incompatibility and geographic isolation (both of which can be involved with speciation), which are discussed in detail below.

**Figure 4:**
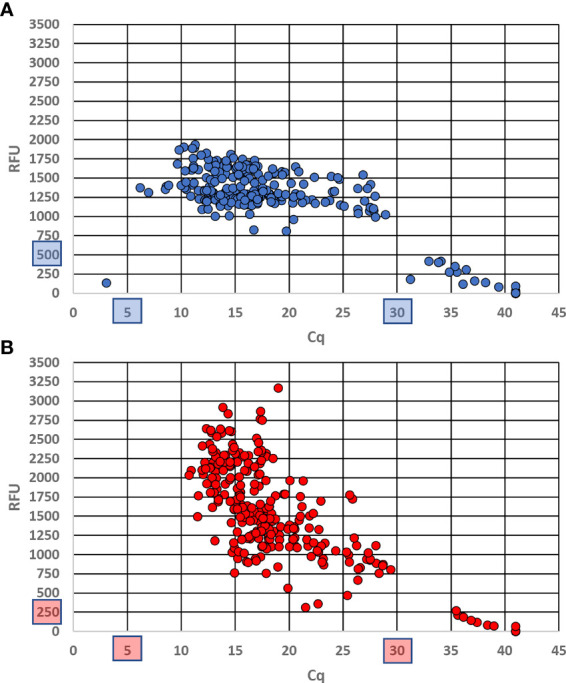
End relative fluorescence unit (RFU) values plotted against Cq values for each probe run in duplex across all replicates using DNA from museum specimens and intercepted *Diabrotica*. **(A)** Results from the CO1 diagnostic probe labeled with a 56-FAM fluorophore; **(B)** results from the 18S control probe labeled with a 5Cy5 fluorophore. Cutoff values are shown in blue **(A)** and red **(B)** boxes.

Of the 110 target *D. undecimpunctata* samples tested with the real-time assay described here, four produced anomalous results. For these anomalous samples, very low (1.6–5.6) and very high (32.9–37.1) C_q_ values were produced for the diagnostic and control probes, respectively. Such an outcome is most likely the result of abnormal differences between CO1 and 18S copy number. Fortunately, in these rare cases the probes can be rerun in separate reactions to confirm the results. Of the 10 *D. undecimpunctata* samples (not included in the 110 target samples) that had been parasitized by *Centistes*, only one produced an anomalous result, which was nearly identical to that of the four samples described above. The non-target *D. balteata* (2), *Lyctus africanus* (1), and early-diverging *D. undecimpunctata* (2) from western California all produced positive results for the control probe and negative results for the diagnostic probe. A third *D. balteata* sample failed for both probes, likely due to deteriorated DNA. All samples that showed amplification for each probe were used to set the relative fluorescence unit (RFU) threshold above which a sample is considered positive. This value was set to 500 for the diagnostic probe and to 250 for the control probe. For each, a C_q_ between 5 and 30 was considered positive. Background values originated from either NTC samples or non-targets in the case of the diagnostic probe and were used to inform threshold setting. No non-target samples produced amplification of the diagnostic assay. Using these thresholds, the C_q_ values across all targets ranged from 6.18 to 28.93 (mean 15.71 ± SD 4.38) for the diagnostic probe (**Figure 4A**) and from 10.72 to 29.41 (mean 17.18 ± SD 3.99) for the control probe (**Figure 4B**). The end RFU values across all replicates ranged from 813.94 to 1934.33 (mean 1359.33 ± SD 209.07) for the diagnostic probe (**Figure 4A**) and 315.21 to 3169.02 (mean 1533.68 ± SD 546.79) for the control probe (**Figure 4B**). In addition, ΔC_q_ (|control probe C_q_ – diagnostic probe C_q_|) was calculated for each reaction containing target DNA using the thresholds described above for which values ranged from 0.01 to 6.56 (mean 1.50 ± SD 0.65). From this, the ΔC_q_ cutoff to confirm a sample as positive was set to 7.

**Figure 5:**
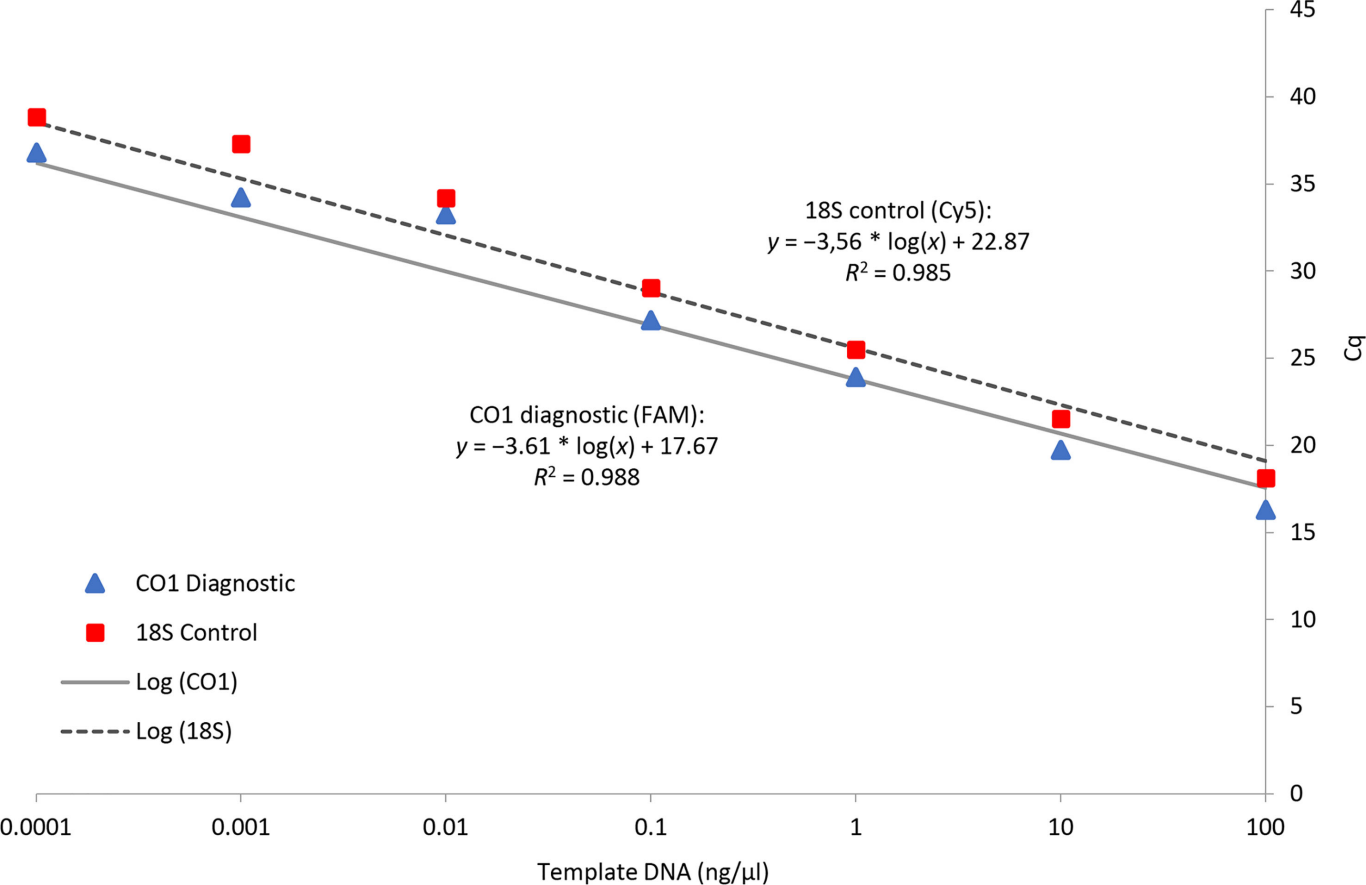
Serial dilutions of template DNA for three *D. undecimpunctata* individuals with a standard curve based on response in Cq for the CO1 diagnostic and 18S control probes run in duplex.

The real-time PCR assay, when run in duplex, showed a linear dose response in C_q_ for both probes across a DNA dilution series (**Figure 5**). From the standard curve, the assay developed here should provide reliable target detection at DNA concentrations ≥ 0.1 ng/µL.

## 4 Discussion

A set of *Diabrotica* CO1 sequences originating from multiple different public data repositories and studies, as well as sequences newly generated as part of this study from museum specimens and port-of-entry interceptions, showed no clear association with current subspecies designations and genetic divergence when multiple phylogenetic approaches were applied (**Figure 1**). The nuclear ITS2 sequence data did not provide any additional resolution among these subspecies; however, like the CO1 data, ITS2 separated all *D. undecimpunctata* samples from other *Diabrotica* species and resolved an outlier lineage from western California (**Supplementary Figures 4–6**). While complete genome data may provide additional resolution (48) and further insight into the relationships among *D. undecimpunctata* lineages, such data cannot be rapidly or inexpensively generated, nor will the level of sampling be equivalent to the currently available CO1 databases for some time. Additionally, CO1 data have proven to be highly effective in the identification of insect species that were originally described based on morphology when applying a monophyly criteria (49, 50). While CO1 is often congruent with morphology-based species descriptions, such data have also been useful in detecting cryptic insect lineages (51–53). Thus, our CO1 and ITS2 results are not intended to immediately refute the validity of the current morphology-based *D. undecimpunctata* subspecific categories, but rather (1) to provide a framework for the re-examination of morphological and biological attributes in these taxonomic categories (54, 55) and (2) to supply data for the development of a molecular assay for use in inspection of transshipped commodities or high-throughput field surveys.

We compared our CO1 data for *D. undecimpunctata* sampled at the subspecific level to *D. virgifera*, the only other *Diabrotica* species with extensive mtDNA sampling at the subspecific level, to examine how genetic and taxonomic patterns correlated within and between these lineages (**Figures 1**, **3**). In both species, subspecific designation did correspond to well-supported CO1 lineages, although more so in *D. virgifera*. The subspecific split between *D. v. virgifera* and *D. v. zeae* is thought to be largely driven by the infection of *D. v. virgifera* males by *Wolbachia* bacteria, resulting in cytoplasmic incompatibility when they mate with uninfected *D. v. zeae* females and thus the perpetuation of a unidirectional reproductive barrier (56). The divergence resulting from this prolonged reproductive incompatibility is evident in the matrilineal CO1 data (**Figure 3**), with the resolution of a *D. v. zeae* lineage restricted to Mexico (save two *D. v. virgifera* from Croatia, which might be the result of misidentification or post-invasion mitochondrial introgression from the loss of *Wolbachia* infection) and a polyphyletic lineage of *D. v. virgifera* and *D. v. zeae* found in both Mexico and the USA. All *D. v. zeae* in the polyphyletic lineage are from either Texas or Mexico, an area which is thought to be a hybrid zone given the intermediacy of traits found among individuals collected in this region (57). As such, the polyphyly in this lineage may be the result of reciprocal crosses between *D. v. virgifera* females and *D. v. zeae* males in which rates of viability are known to be much higher (56). A third early-diverging lineage of *D. v. zeae* from Guatemala was resolved in the dataset and may represent an ancestral haplotype, as it shares several nucleotide variants with outgroup taxa not present in the other two lineages (**Supplementary Data Sheet 3**). This third, evidently relictual, lineage may in part be the result of mitochondrial displacement induced *via Wolbachia*-driven cytoplasmic incompatibility (58, 59), geographic isolation (60), and/or positive selection (61). By extension, the early-diverging lineage resolved in *D. undecimpunctata* may have evolved *via* similar processes given similarities in genetic and geographic patterns between these congeneric lineages, but more work is needed to assess what factors are driving these patterns of asymmetrical mitochondrial haplotype counts in these *Diabrotica* lineages. Furthermore, the question of whether these early-diverging monophyletic lineages should be treated as distinct species needs additional research. Interestingly, the species range of *D. u. undecimpunctata* has been considered to be restricted to the west coast of the USA and it has also been considered the most morphologically divergent and easily identified subspecies (20). Beyond the taxonomic need to study these early-diverging *Diabrotica* lineages, phylogeographic and ecological studies may provide additional insights into the relictual status and biology of these lineages. For instance, if Guatemala and western California were the locations in which *D. virgifera* and *D. undecimpunctata* first evolved, then the dispersal of *D. undecimpunctata* may not have initially followed maize cultivation from Mexico as was the case for *D. virgifera* (26). Study of *Diabrotica* populations in their centers of origin/diversity may also yield insights into natural enemies or other aspects of their biology applicable to controlling their populations in agroecosystems [e.g. (62)].

Like the early-diverging CO1 lineages found in *D. virgifera* and *D. undecimpunctata*, several similar patterns of divergence were noted in our analyses of CO1 from other *Diabrotica* taxa; these may have been the result of operational error and/or biological processes. For example, within the species *D. adelpha* and *D. limitata*, haplotypes were separated by 53 and 28 SNVs, respectively (**Figure 2**). The publicly available CO1 sequences for *D. biannularis*, *D. balteata*, *D. speciosa*, and *D. tibialis* were, by contrast, similar to what is generally observed for intraspecific variability in beetles and other insects (49, 63), although increased sampling is needed to more fully describe the nucleotide variability in these *Diabrotica* lineages. Given the discrepancies observed between taxonomic designation and CO1 haplotypes, both here and in other phylogenetic appraisals of *Diabrotica* and allied species (22), a concerted effort should be made to better understand how the sorting and evolution of mitochondrial and other genetic and genomic data corresponds to morphological, behavioral, and adaptive divergence. Such efforts will provide an evolutionary context to the study of agriculturally important attributes of *Diabrotica* such as host preferences, xenobiotic metabolism, and natural and anthropogenic patterns of dispersal and migration.

The real-time PCR assay described here was effective at distinguishing *D. undecimpunctata* from related species. Given the known challenges of separating some *D. undecimpunctata* subspecies on the basis of morphology and the frequency with which these beetles are intercepted on agricultural commodities, the real-time PCR assay presented here represents a useful tool for rapid identification at ports of entry. Similarly, given concerns about the introduction of *D. undecimpunctata* outside North America, this assay could also be used for prescreening of agricultural commodities before export. The assay was designed to exclude the early-diverging western California lineage at present, until follow-up work can clarify the species status of these individuals; however, given that these samples are rare and geographically isolated, this should not affect routine screening efforts. If this haplotype needs to be included in future assays, design of a probe should be straightforward given the number of SNVs separating this haplotype from the next-nearest specimens.

When other *Diabrotica* species were tested with our real-time PCR assay there was no signal for the diagnostic probe, indicating that these samples did not produce off-target amplification. In a small number of target samples (five), large differences between diagnostic and control probe C_q_ might have been related to early stages of *Centistes* parasitism, as one of these samples was known to have been parasitized (47), or to other factors known to affect CO1 copy number (64). In the future, using only legs in DNA extraction might limit such imbalances in CO1 and 18S copy number to the extent that these were associated with the whole-body extractions employed throughout this study. Testing of more *Diabrotica* species and of *D. undecimpunctata* at different life stages should be carried out to further validate the findings reported here. A broader diversity of sample types should also be tested with the primers and probes developed for this study so that this assay can be adapted for other applications, such as testing of soil samples for the presence of *D. undecimpunctata* larvae [e.g. (65)] or the processing of bulk samples using ddPCR [e.g. (66)]. Soil sampling of this type could be an important planning tool for producers, helping them to decide to plant resistant crops when evidence of *D. undecimpunctata* is found. The assay described here could be expanded to identify not only *D. undecimpunctata* but also associated plant pathogens such as *E. tracheiphila* in multiplex PCRs.

## Data availability statement

The datasets presented in this study can be found in online repositories. The name of the repository and accession numbers are as follows: NCBI; OQ641612–OQ641622 and OQ649623–OQ649739.

## Author contributions

FZ, LT, and TG conceived and designed the study. CW, FZ, and LT prepared and processed the samples used in the study. CW, FZ, and LT performed analyses. LT and TG acquired funding and administered the project. AK, FZ, CW, LT, and TG wrote the original draft of the manuscript and LT, CW, FZ, AET, TG, AK, and AKT helped to write and edit all subsequent versions. All authors contributed to the article and approved the submitted version.

## Footnotes

1
https://www.geneious.com.

2
https://www.idtdna.com/calc/analyzer.
