# Supplementary material for: CO1 barcodes resolve an asymmetric biphyletic clade for Diabrotica undecimpunctata subspecies and provide nucleotide variants for differentiation from related lineages using real-time PCR
Source: Front Insect Sci. 2023 Apr 20;3:1168586. doi: 10.3389/finsc.2023.1168586 (PMC10926502; doi:10.3389/finsc.2023.1168586)
Supplement: Supplementary file 4 [file DataSheet_4.pdf]

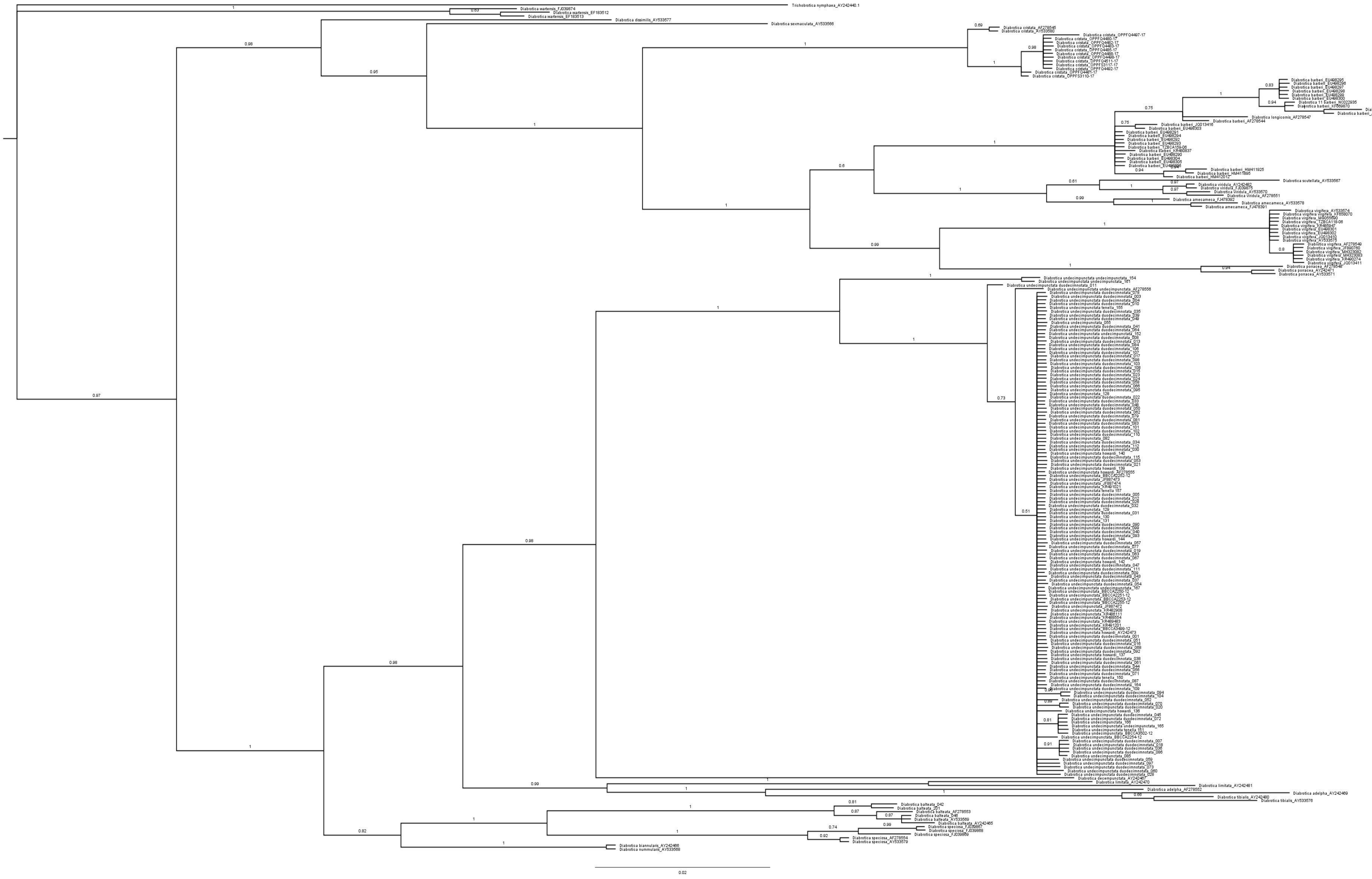

Supplementary Figure 1 A phylogenetic tree for *Diabrotica* using Bayesian inference with a JC69 substitution model on an alignment of 227 CO1 DNA barcodes. Values at nodes are posterior probabilities. *Trichobrotica nymphaea* was set as an outgroup.

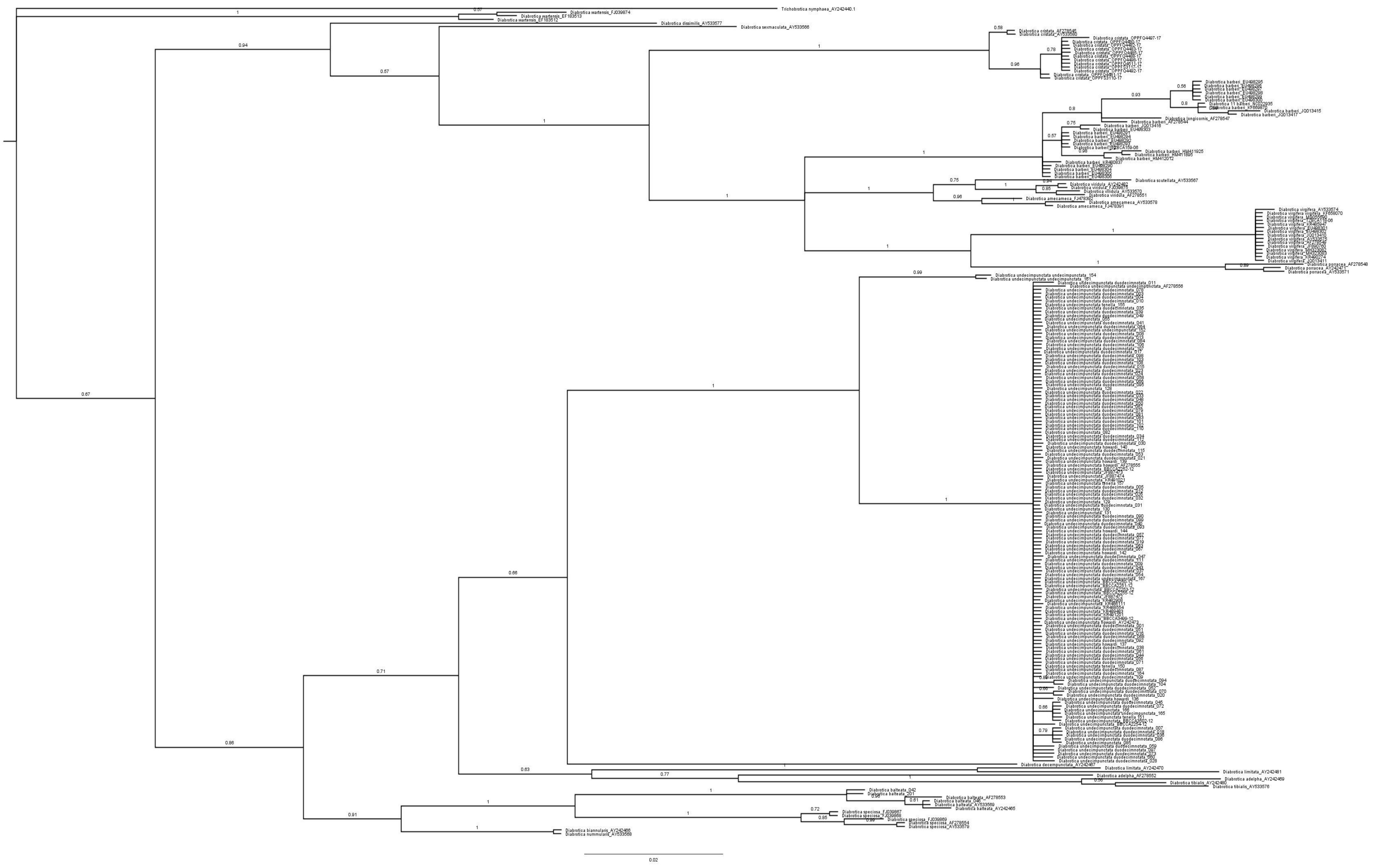

Supplementary Figure 2 A phylogenetic tree for *Diabrotica* using Bayesian inference with a GTR substitution model on an alignment of 227 CO1 DNA barcodes. Values at nodes are posterior probabilities. *Trichobrotica nymphaea* was set as an outgroup.

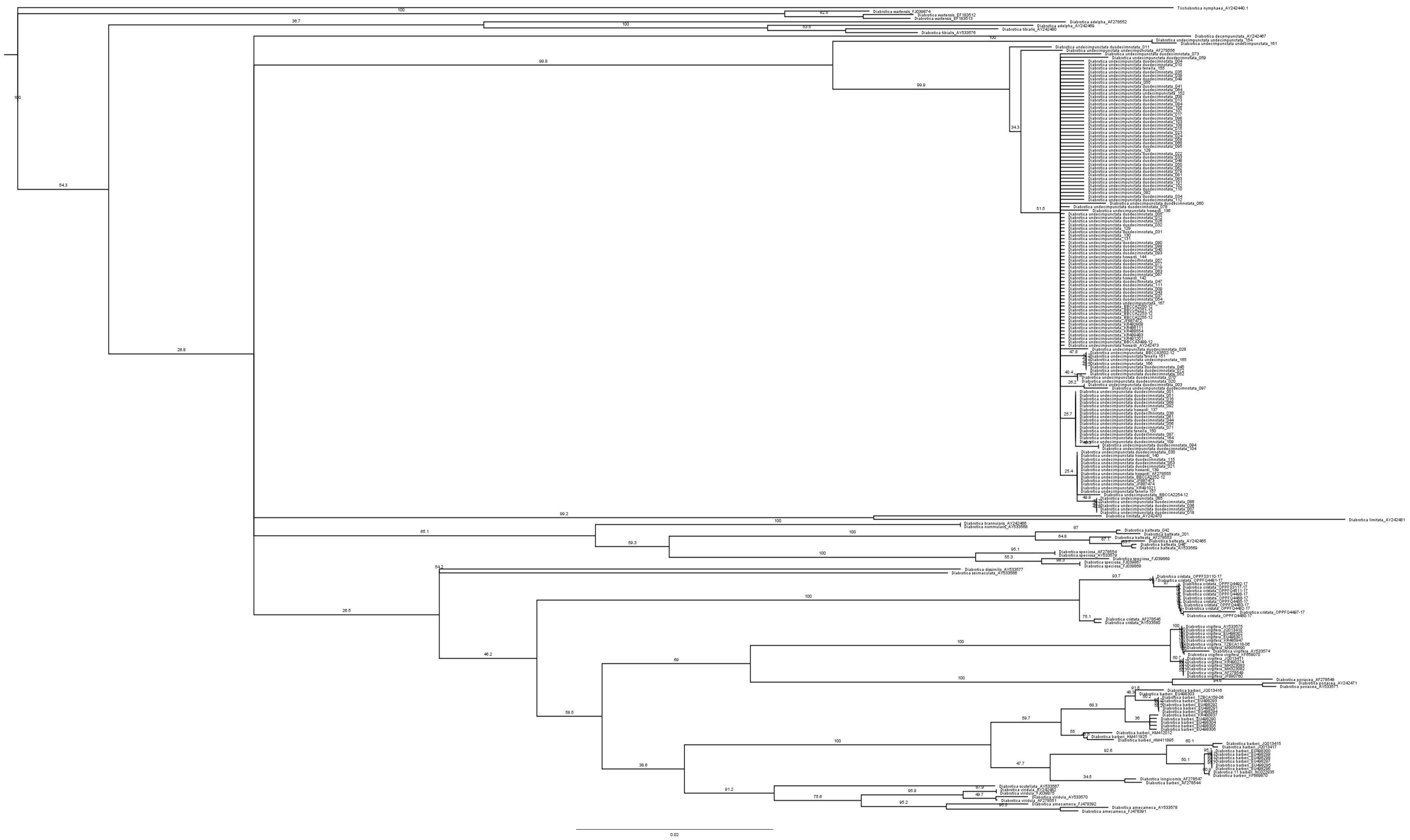

Supplementary Figure 3 A Neighbor Joining tree for *Diabrotica* using the Tamura-Nei genetic distance model on an alignment of 227 CO1 DNA barcodes. Values at nodes are Jackknife support. *Trichobrotica nymphaea* was set as an outgroup.

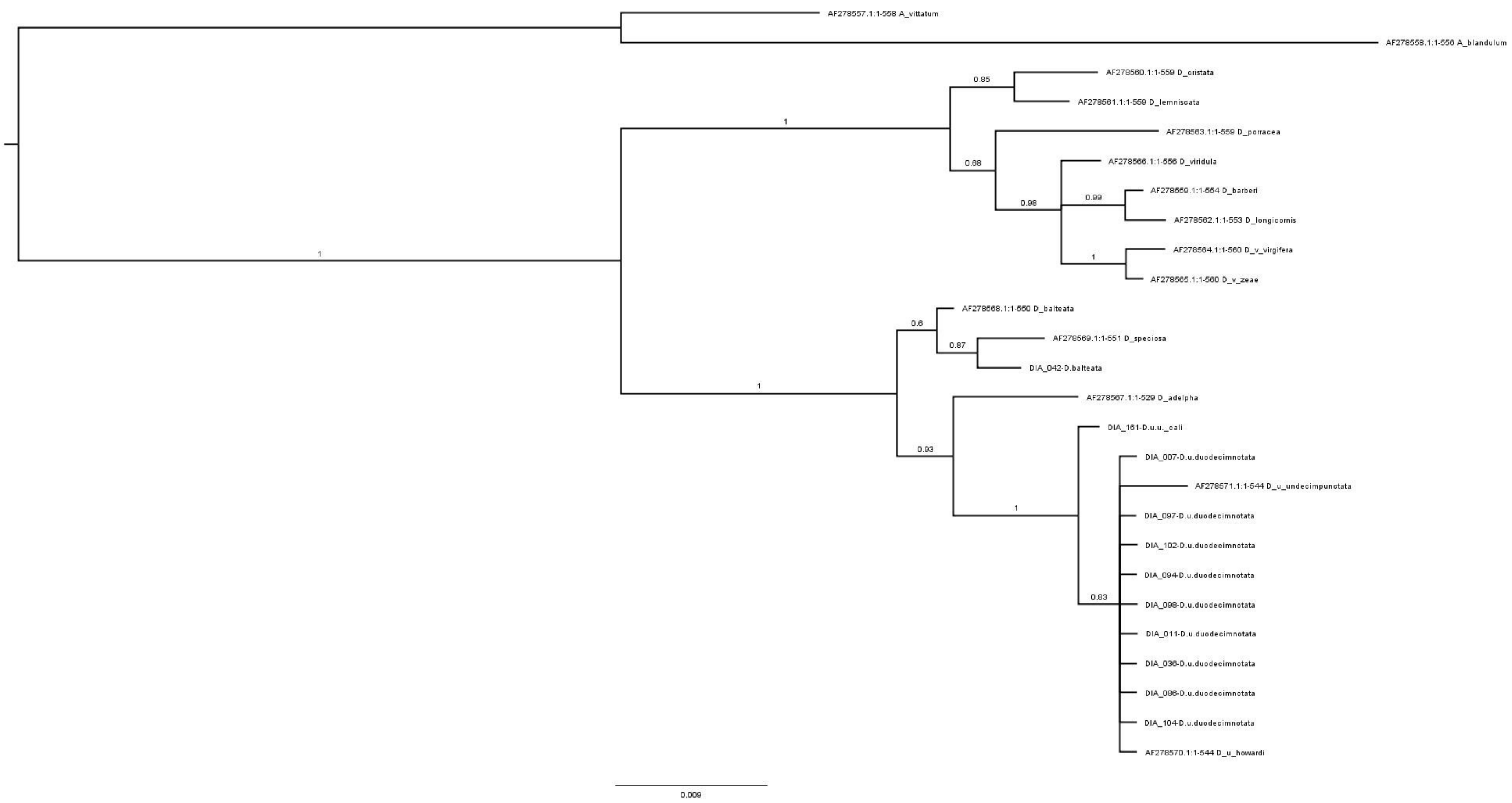

Supplementary Figure 4 A phylogenetic tree for *Diabrotica* using Bayesian inference with a JC69 substitution model on an alignment of 26 ITS2 sequences. Values at nodes are posterior probabilities. *Acalymma vittatum* was set as an outgroup.

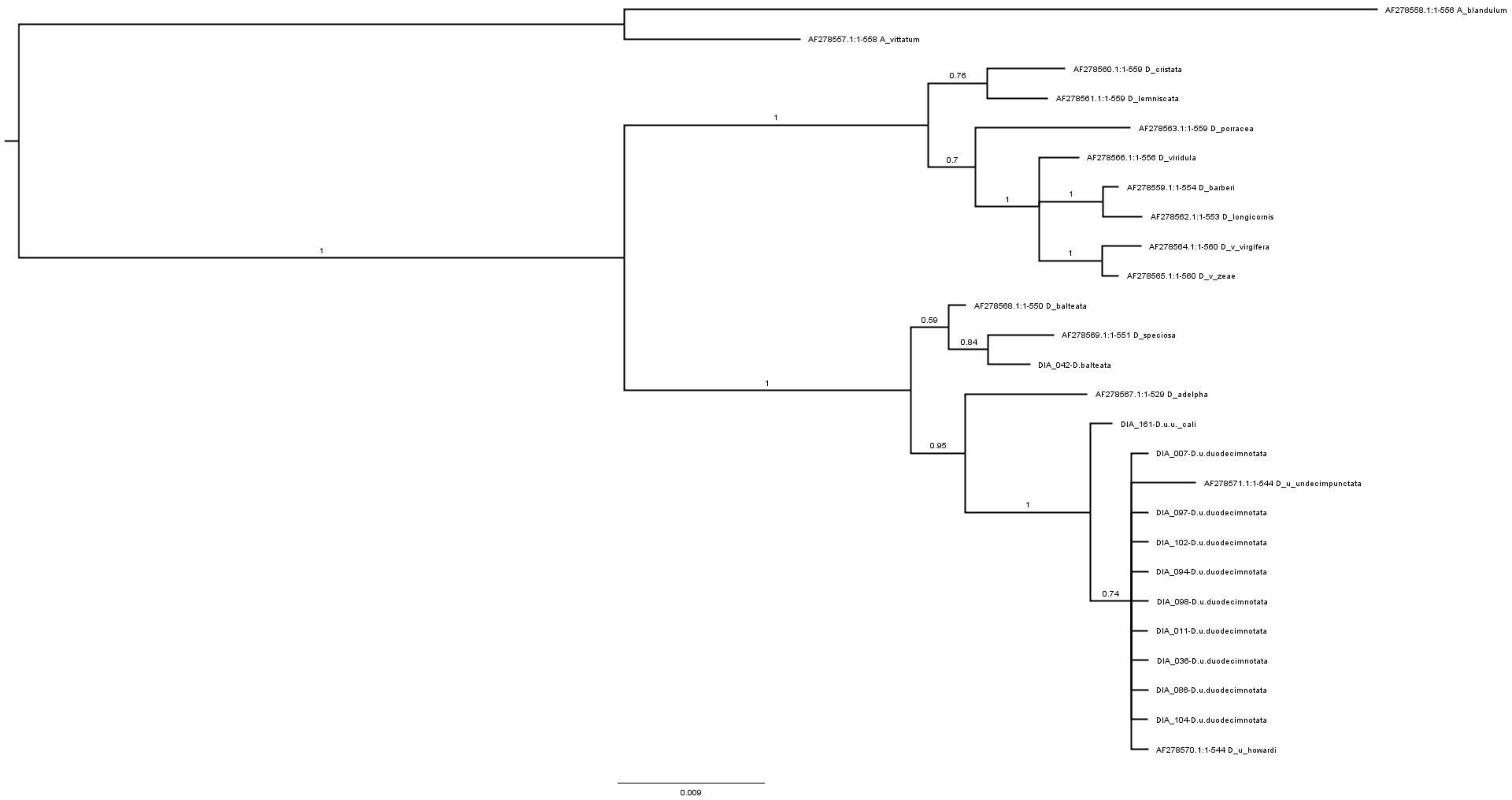

Supplementary Figure 5 A phylogenetic tree for *Diabrotica* using Bayesian inference with a GTR substitution model on an alignment of 26 ITS2 sequences. Values at nodes are posterior probabilities. *Acalymma vittatum* was set as an outgroup.

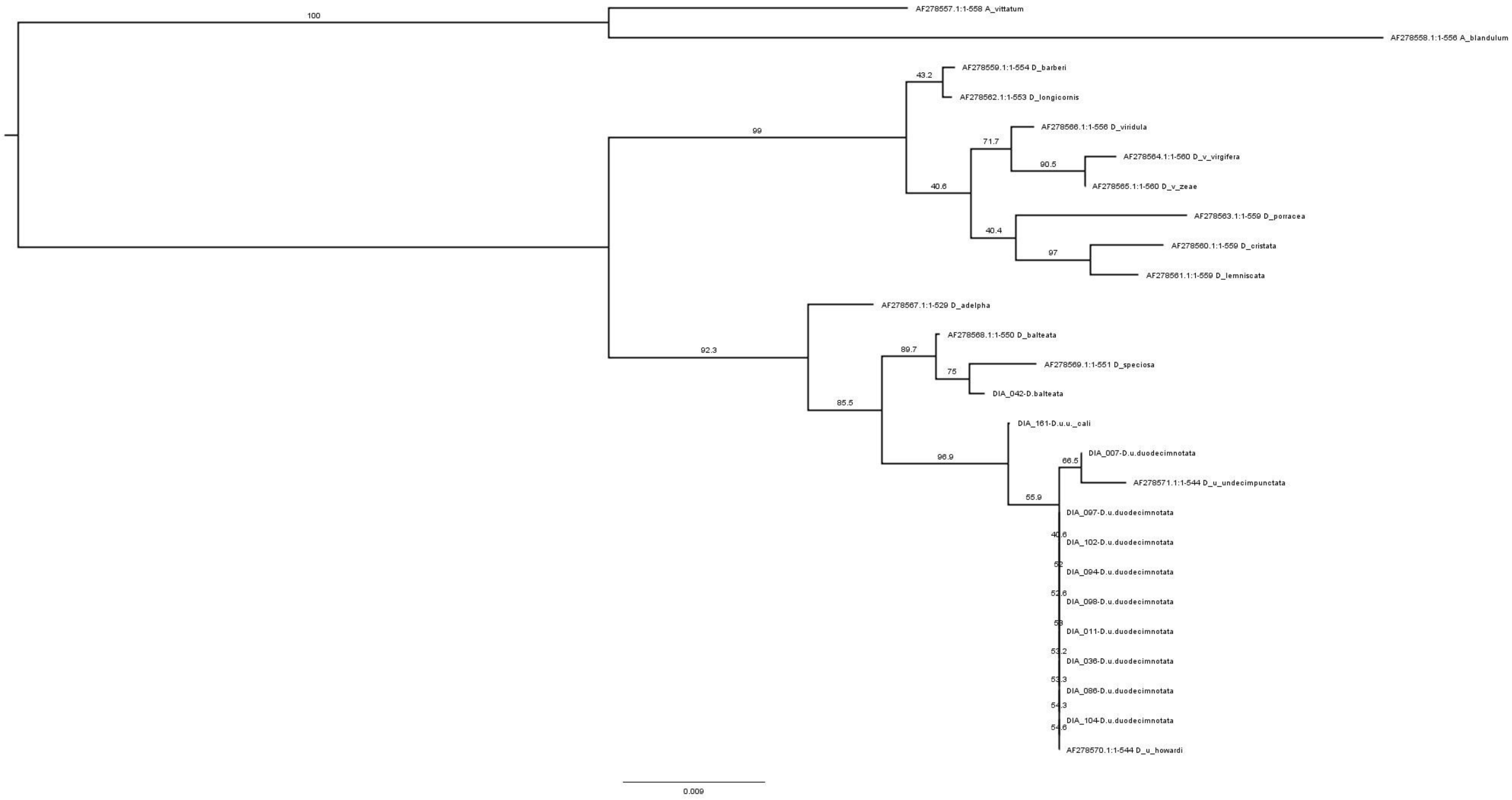

Supplementary Figure 6 A Neighbor Joining tree for *Diabrotica* using the Tamura-Nei genetic distance model on an alignment of 26 ITS2 sequences. Values at nodes are Jackknife support. *Acalymma vittatum* was set as an outgroup.

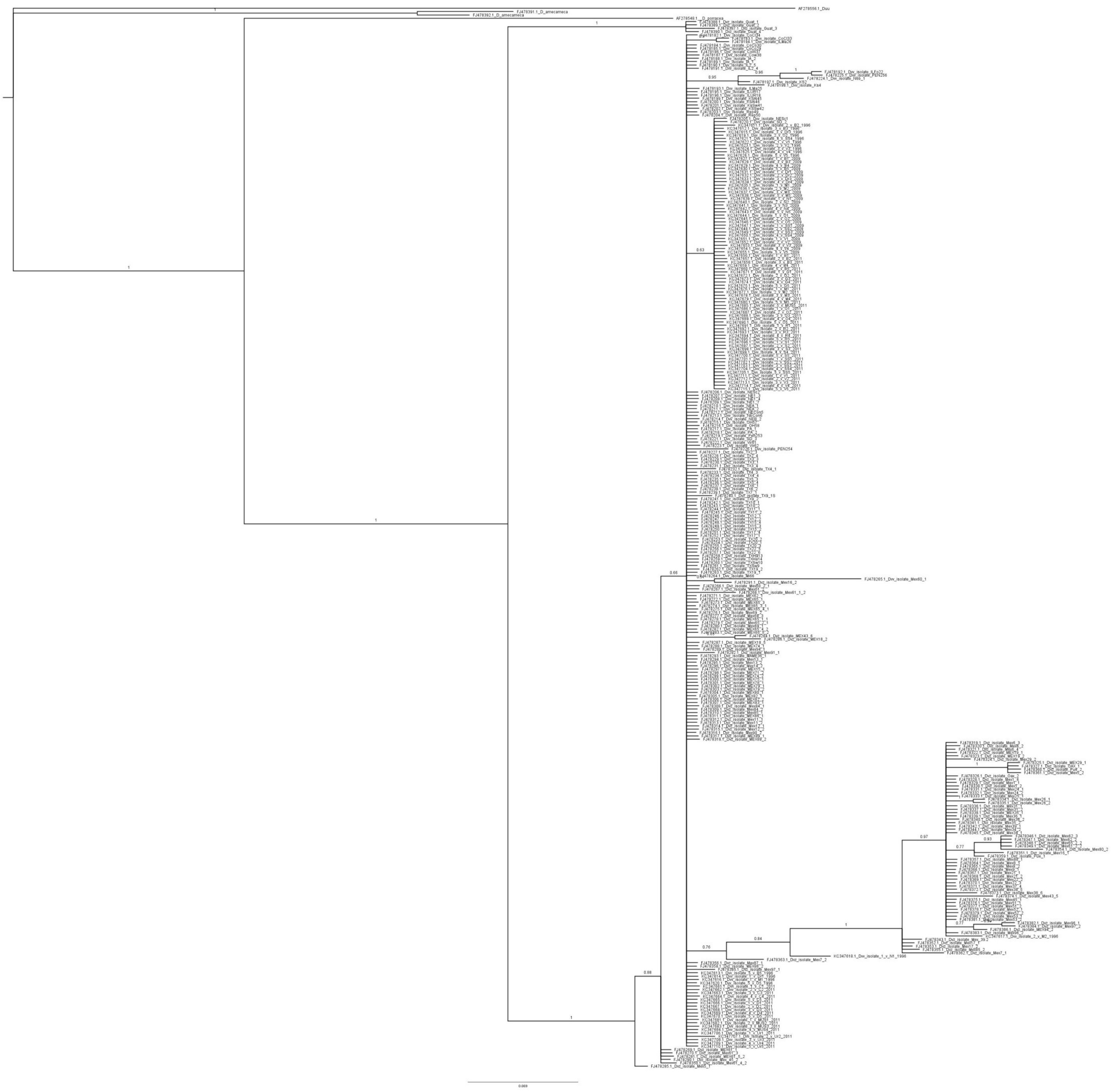

Supplementary Figure 7 A phylogenetic tree for *Diabrotica virgifera* subspecies using Bayesian inference with a JC69 substitution model on an alignment of 318 CO1 DNA barcodes. Values at nodes are posterior probabilities. *Diabrotica undecimpunctata* was set as an outgroup.

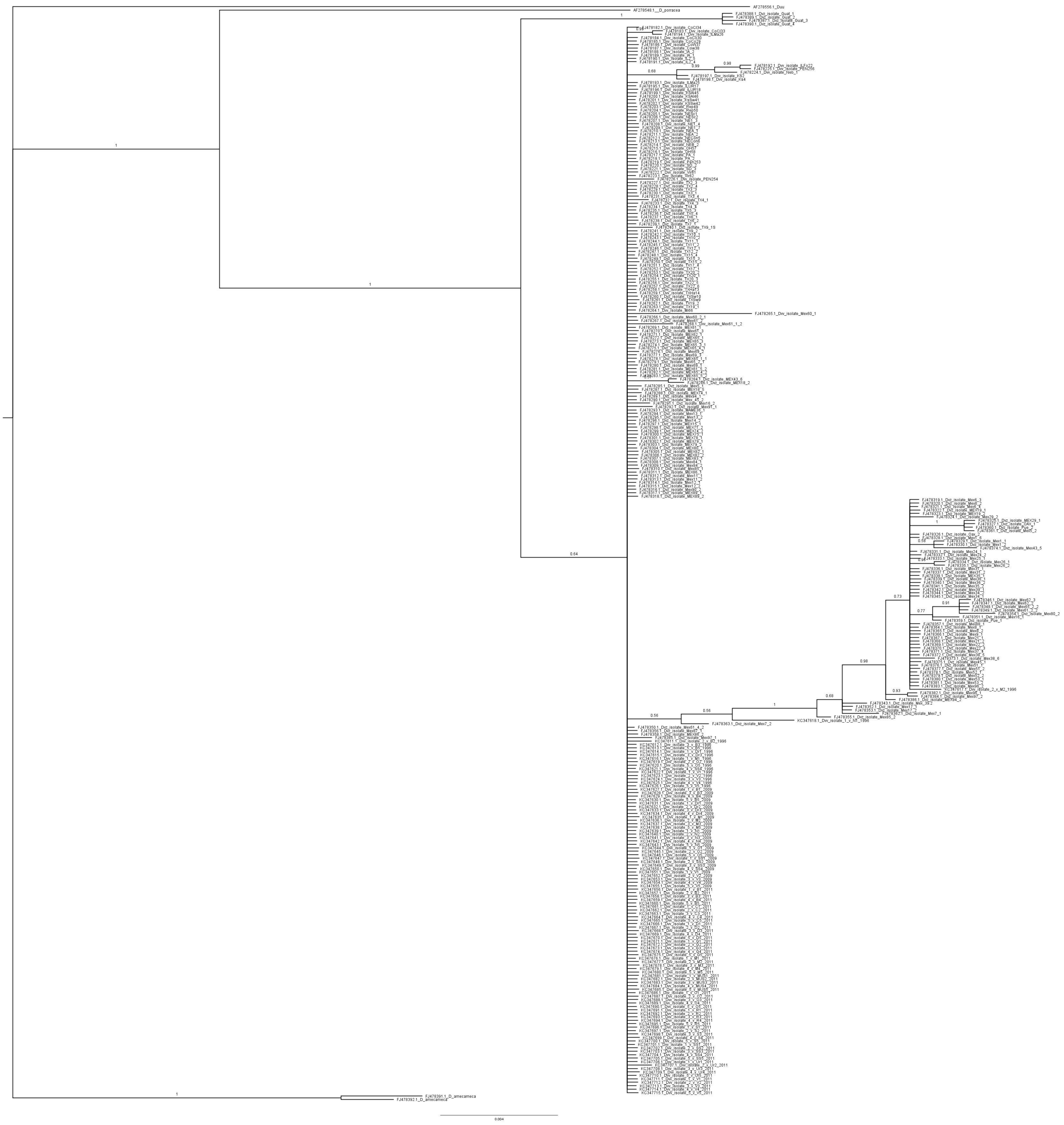

Supplementary Figure 8 A phylogenetic tree for *Diabrotica virgifera* subspecies using Bayesian inference with a GTR substitution model on an alignment of 318 CO1 DNA barcodes. Values at nodes are posterior probabilities. *Diabrotica undecimpunctata* was set as an outgroup.

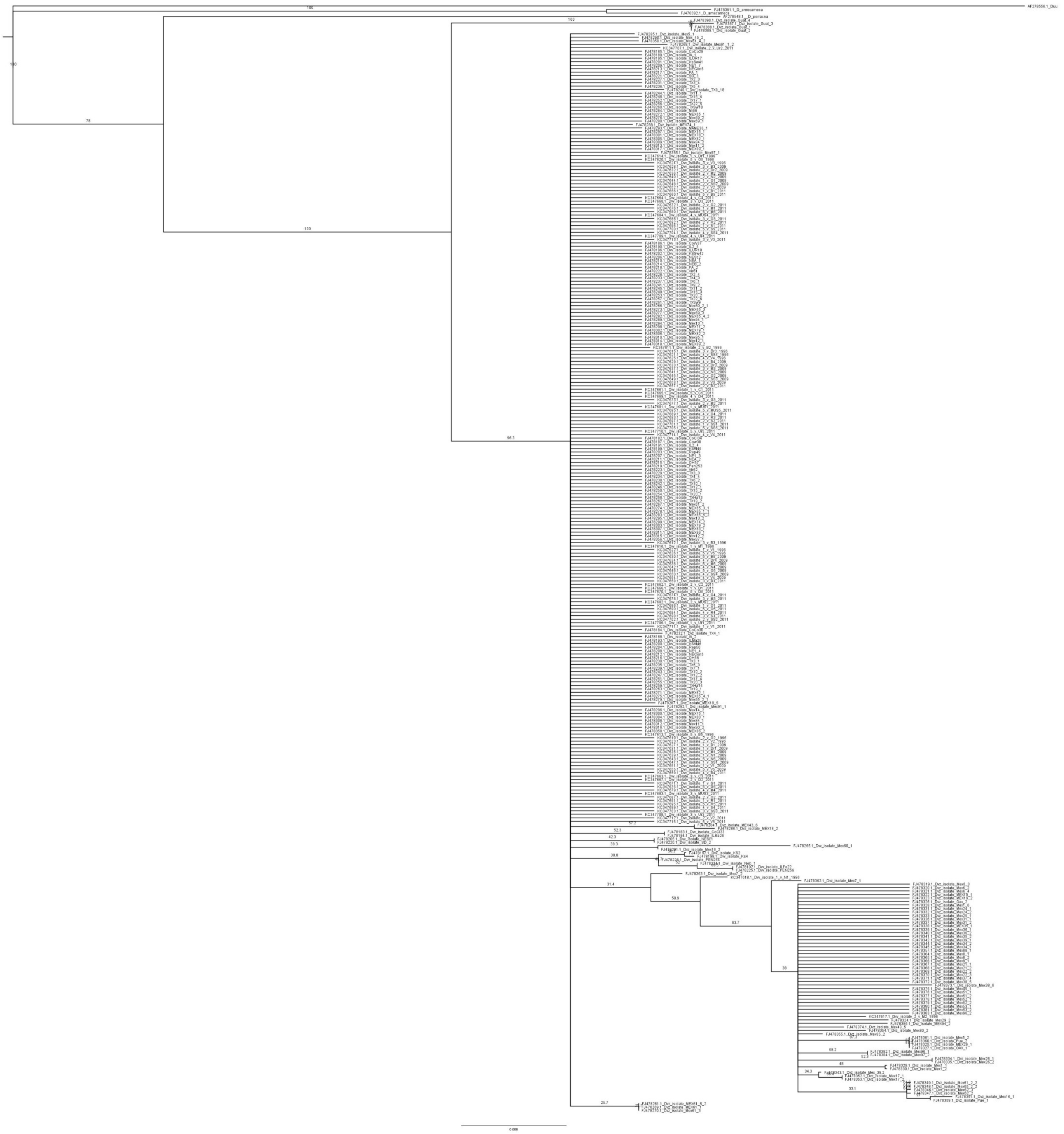

Supplemental Figure 9 A Neighbor Joining tree for *Diabrotica virgifera* subspecies using the Tamura-Nei genetic distance model on an alignment of 318 CO1 DNA barcodes. Values at nodes are Jackknife support. *Diabrotica undecimpunctata* was set as an outgroup.
